# Molecular Mechanisms of Insect Resistance in Rice and Their Application in Sustainable Pest Management

**DOI:** 10.3390/insects17010111

**Published:** 2026-01-19

**Authors:** Dilawar Abbas, Kamran Haider, Farman Ullah, Umer Liaqat, Naveed Akhtar, Yubin Li, Maolin Hou

**Affiliations:** 1 State Key Laboratory for Biology of Plant Diseases and Insect Pests, Institute of Plant Protection, Chinese Academy of Agricultural Sciences, Beijing 100193, China; dilawar72@yahoo.com (D.A.); lc01972@163.com (Y.L.); 2 College of Plant Science and Technology, Huazhong Agricultural University, Wuhan 430070, China; kamranhaider345@gmail.com; 3 Institute of Bio-Interaction, Xianghu Laboratory, Hangzhou 311258, China; 4 Department of Zoology, Wildlife and Fisheries, University of Agriculture, Faisalabad 38000, Pakistan; umer.liaqat@uaf.edu.pk; 5 Department of Zoology, Government Graduate College Chunian, Kasur 55050, Pakistan; naveed.akhtar430@gmail.com

**Keywords:** rice, insect resistance, functional genomics, resistance genes, molecular markers, integrated pest management

## Abstract

Rice is a key food crop worldwide, but its yield and quality are severely constrained by insect pests. As environmental and regulatory restrictions on chemical pesticides grow, developing insect-resistant rice varieties has become a sustainable way to protect food security. This review covers recent progress in functional genomics and molecular marker mapping related to insect resistance in rice. We highlight the identification, cloning, and functional analysis of resistance genes targeting major pests, including the brown planthopper, rice gall midge, white-backed planthopper, small brown planthopper, and rice leaf roller. Several important resistance genes (such as *Bph14*, *Bph3*, and *Bph29*) have been cloned, and their roles in rice immunity have been clarified—covering insect feeding signal recognition, activation of salicylic acid and jasmonic acid pathways, and regulation of MAPK cascades, calcium signaling, and reactive oxygen species production. We also discuss how molecular marker-assisted selection, gene pyramiding, and transgenic techniques are used in modern rice breeding. Finally, we address future challenges and opportunities, stressing the importance of utilizing wild rice germplasm, understanding insect effector–plant immune interactions, and applying molecular design breeding to create long-lasting insect-resistant rice varieties that can withstand changing pest pressures and climate conditions.

## Simple Summary

Insect pests are a major cause of yield loss in rice, and breeding insect-resistant varieties is an effective way to reduce reliance on chemical pesticides. This review summarizes recent progress in understanding how rice plants resist insect pests such as the brown planthopper, rice gall midge, and white-backed planthopper. We highlight key resistance genes and explain how rice recognizes insect attacks and activates defense responses using plant hormone signaling pathways, including the salicylic acid and jasmonic acid pathways. The review also covers modern breeding techniques like molecular markers, gene pyramiding, and transgenic technologies, along with the significance of wild rice species as resistance sources. Overall, this work aims to support the development of durable, insect-resistant rice varieties and promote sustainable rice production amid changing pest pressures and climate conditions.

## 1. Introduction

Rice is one of the most essential food crops worldwide, with over half of the global population relying on it as a staple diet. Rice was domesticated and cultivated in the river valleys of China, South Asia, and Southeast Asia around 10,000 years ago, with the earliest records dating back to approximately 3000 BC in China [1]. Most rice is grown in humid environments, which promote the development and spread of insect pests.

More than 30 insect pest species attack rice crops and can cause significant yield losses [2]. These insects feed on rice throughout its life cycle, from the seedling stage to maturity, and damage different parts of the plant. In addition to their feeding stage and location, rice variety and cultivation practices strongly influence insect population dynamics [3]. Recent studies show that insect pests remain a major obstacle to global rice production. Experts estimate that pests and pathogens, including insects, can reduce global rice yields by about 30% (roughly 25–41%) in major production regions. Field studies further reveal that key rice pests, such as planthoppers, stem borers, and gall midges, can cause average yield losses of 10–50% or more, depending on pest pressure and management strategies, with severe outbreaks causing even greater losses in some areas [4]. In the Philippines, rice yield losses over the past 13 years have ranged from approximately 12.7% in irrigated fields to 5–71% in upland paddies and 2–88% in wetland paddies [4]. Recently, brown planthopper outbreaks have reduced rice yields by about 10% in China, India, Indonesia, the Philippines, Thailand, and Vietnam, contributing to rising food prices since 2003 [5]. Therefore, reducing pest damage is crucial for improving rice yields and ensuring production stability.

Insecticides have been widely used to control insect pests in rice production and have played a key role in reducing yield losses. However, increasing evidence shows that the intensive and long-term reliance on chemical insecticides raises many concerns that go beyond just sublethal effects on non-target organisms. These concerns include the quick development of insecticide resistance in major rice pests, pest resurgence, and secondary pest outbreaks caused by disrupting natural enemy communities, environmental contamination of soil and water, and potential health risks to humans [6,7,8]. Additionally, stricter regulations and public concern over pesticide residues further limit the sustainability of chemical pest control [7,8]. Overall, these challenges emphasize the importance of finding alternative and complementary pest management methods, especially the development and use of insect-resistant rice varieties as an environmentally friendly and long-lasting solution.

Given these limitations of chemical control, host plant resistance has emerged as a key element of sustainable rice pest management. Plant resistance to insects can greatly reduce damage caused by pests and can be inherited by future generations [9]. Growing insect-resistant rice varieties decreases pest-related yield losses without adding extra burden on farmers, making this method practical and widely used. Such varietal resistance aligns well with integrated pest management techniques, including healthy cultivation, biological control, and careful pesticide use. As a result, enhancing crop insect resistance is recognized as an important global trend and a vital strategy for pest management in China [10]. Insect-resistant rice directly impacts pest survival and behavior, whereas susceptible rice often relies heavily on chemical pesticides. In resistant rice varieties, pest physiology and behavior are affected; for example, the survival and migration abilities of brown planthoppers are significantly diminished [11]. Therefore, developing rice varieties resistant to multiple pests remains a primary goal of rice breeding, as it can lower pesticide use and reduce pest-related yield losses [4].

This review aims to systematically summarize recent advances in the identification, mapping, and cloning of insect-resistance genes in rice, synthesize current knowledge of the molecular mechanisms behind rice–insect interactions, and discuss modern breeding strategies for developing durable insect-resistant rice varieties. The review is organized to first introduce insect-resistant germplasm resources and gene mapping, followed by an overview of cloned resistance genes and their molecular mechanisms. Finally, it examines physiological responses, breeding applications, and future prospects for sustainable rice pest management.

## 2. Insect-Resistant Rice Germplasm Resources

In this review, “wild rice” refers to wild *Oryza* species, whereas cultivated landraces and locally adapted, unimproved materials are referred to as *Oryza sativa* germplasm. Rice germplasm resources are broadly classified into cultivated rice and wild rice species. Cultivated rice includes African rice (*O. glaberrima*) and Asian rice (*O. sativa*), with Asian rice further divided into the two major subspecies, indica and japonica. These groups differ in genetic background and levels of insect resistance, but all represent important sources of resistance genes for rice breeding programs. By the end of 1995, the China National Germplasm Bank had collected 64,186 rice varieties, while the International Rice Research Institute had assembled 84,200 rice germplasm accessions. Together, these collections represent important sources of genes for insect resistance [12].

Wild rice species have been exposed to insect pests for long evolutionary periods and have therefore developed diverse and stable resistance traits. Large-scale evaluations conducted by the International Rice Research Institute showed that resistance to brown planthopper biotypes occurs much more frequently in wild rice than in cultivated rice, with resistance levels in wild rice being several times higher [13]. In cultivated rice, indica varieties generally show stronger insect resistance than japonica varieties, and tropical rice germplasm is often more resistant than temperate types. These differences highlight the importance of wild rice species and traditional landraces as valuable genetic resources for improving insect resistance in modern rice breeding [12,14].

In recent years, significant progress has been made in identifying insect-resistant rice germplasm, particularly against the brown planthopper (*Nilaparvata lugens*). Functional genomics studies have led to the cloning of several major resistance genes, including *Bph14*, *Bph6*, and *Bph9*. These genes contribute to resistance by activating plant defense responses, such as strengthening cell walls, regulating immune signaling pathways, and balancing plant growth and defense [13,14]. The jasmonic acid signaling pathway also plays an important role in resistance; for example, activation of OsMYC2 promotes cell wall reinforcement and enhances resistance to both brown and white-backed planthoppers [15]. At the same time, brown planthoppers can evolve virulence factors that weaken plant defenses, emphasizing the need to consider pest adaptation in resistance breeding [16,17].

Marker-assisted selection and gene pyramiding have been widely used to combine multiple resistance genes into elite rice lines, resulting in improved and more stable resistance. Restorer lines carrying combinations of genes such as *Bph6*, *Bph9*, *Bph14*, and *Bph15* have shown enhanced resistance under field conditions, and several resistant varieties have been released for cultivation [18,19]. Despite this progress, wild rice species, especially *Oryza rufipogon*, remain an important source of new resistance genes that may help address emerging pest pressures and climate-related challenges [20]. To support the effective use of resistant germplasm, several standardized screening methods have been developed to evaluate rice resistance at both seedling and adult stages. Greenhouse screening allows for the rapid identification of resistant materials, which are then validated under field conditions. The International Rice Research Institute developed a standard evaluation system that scores plant damage on a scale from 0 (no damage) to 9 (severe damage), allowing quantitative comparison of resistance levels among rice genotypes [12,21]. This system has been widely adopted and provides a reliable basis for selecting insect-resistant rice germplasm.

Researchers have screened a wide range of insect-resistant germplasm in both cultivated and wild rice. Wild rice has evolved under long-term insect pressure and therefore contains abundant and diverse resistance resources. Screening studies conducted by the International Rice Research Institute further demonstrated that wild rice species harbor resistance to brown planthopper biotypes at a substantially higher frequency than cultivated rice, with resistance detected approximately 30-fold more often and multi-biotype resistance exceeding 50-fold. This reinforces the importance of wild germplasm as a critical reservoir of durable resistance traits [22]. These results suggest that wild rice may possess a broader resistance spectrum than cultivated rice, and that indica and tropical cultivars tend to exhibit higher insect resistance than japonica and temperate types. These germplasm resources provide an important genetic basis for improving insect resistance in rice breeding.

## 3. Mapping of Insect-Resistance Genes in Rice

To develop insect-resistant rice and clone resistance genes, researchers have introduced resistance traits from traditional rice varieties and wild rice species into modern cultivated lines. The transfer of these genes requires careful consideration of genomic compatibility. 

Wild rice species with the AA genome, such as *Oryza rufipogon*, *O. nivara*, and *O. barthii*, can be directly crossed with cultivated rice (*O. sativa*) to introduce insect-resistance genes. In contrast, wild rice species with non-AA genomes, including *O. officinalis* (CC), *O. minuta* (BBCC), and *O. australiensis* (EE), are reproductively isolated from cultivated rice and require specialized techniques, such as embryo rescue or bridge crosses, to overcome hybridization barriers [23]. Introgression lines are mainly developed through hybridization, backcrossing, and embryo rescue, and different mapping populations, including recombinant inbred lines (RILs), F_2_, F_3_, and backcross (BC) populations, are used to identify insect-resistance genes. Using these approaches, many resistance genes and quantitative trait loci (QTLs) have been mapped onto molecular marker-based genetic linkage maps, providing valuable resources for breeding insect-resistant rice varieties.

### 3.1. Mapping of Nilaparvata lugens-Resistance Genes in Rice

**Figure 1:**
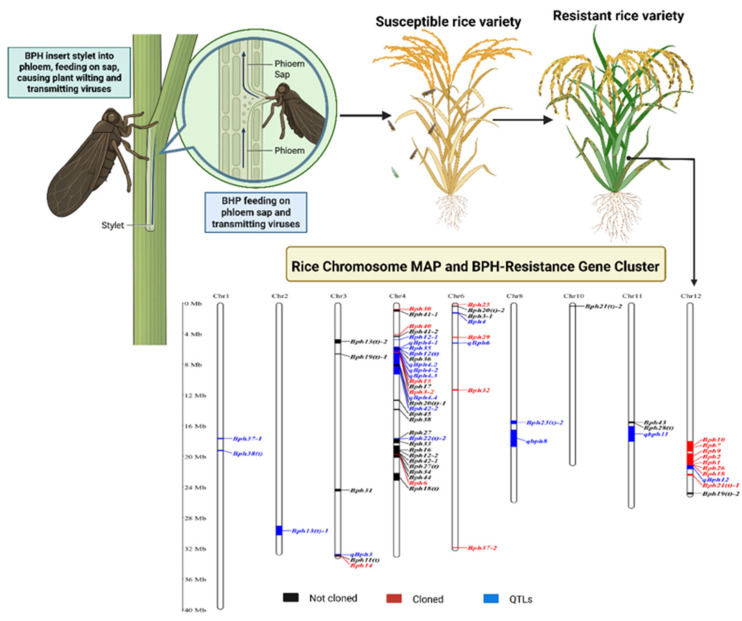
Rice BPH-resistant genes/QTLs distribution on chromosomes. Overall, 70 genes/QTLs were identified in rice. Except for *Bph-5*, *Bph-8 (bph8)*, *Bph-23(t)-1. Bph-24(t) {bph-24(t)}. Bph-39(t) bph-39(t)* and *Bph-40(t)-1 {bph-40(t)}*, the leftover genes/QTLs (44 genes and 20 QTLs) were mapped on chromosomes 1, 2, 3, 4, 6, 8, 10, 11, and 12, respectively. Black typeface designates genes that have been mapped but not yet cloned; red indicates cloned genes, and blue represents QTLs. Adopted from the [25].

The brown planthopper (*Nilaparvata lugens* Stål) is one of the most serious pests of rice, feeding on phloem sap through its piercing–sucking mouthparts. In many countries, direct feeding damage and virus transmission by this pest cause rice yield losses exceeding 10%. Since *N. lugens* has become resistant to insecticides in many areas, planting plant-hopper-resistant rice varieties is the primary method for controlling *N. lugens*. Since the *Nilaparvata lugens*-resistant gene *Bph1* was first identified in cultivated rice, significant progress has been made in understanding brown planthopper (BPH) resistance [24]. In 1971, the International Rice Research Institute (IRRI) identified and mapped the first BPH-resistant gene, *Bph1*. Since then, a total of 70 BPH-resistant genes and quantitative trait loci (QTLs), including 50 genes and 20 QTLs, have been reported in rice. These resistance loci are mainly found on chromosomes 3, 4, 6, and 12 (Figure 1; Supplementary Table S1). To prevent confusion caused by repeated gene names related to BPH resistance, a standardized naming system based on the order of gene discovery has been applied. Notably, most mapped BPH-resistant genes are clustered on chromosomes 3, 4, 6, and 12.

Ten resistance genes, including *Bph-1*, *Bph-2*, *Bph-7*, *Bph-9*, *Bph-10*, *Bph-18*, *qBph-12*, *Bph-19(t)-1*, and *Bph-26*, are clustered between the molecular markers RM17 and RM7102 on chromosome 12. In addition, chromosome 4 contains three major regions harboring about 30 resistance genes. The first region (0.90–1.10 Mb), located between markers SWRm_01522 and SSR28, includes *Bph-30* and *Bph-41-1*. The second region, between markers YM190 and W4_4_3, contains *Bph40-2*, *Bph12-1*, *Bph41-2*, *Bph-35*, *qBph4-1*, *Bph36*, *Bph12(t)*, *qBph4-2*, *qBph4.2*, *qBph4.3*, *Bph-15*, *Bph-17*, *Bph3-2*, *qBph4.4*, *Bph-20(t)-1*, *Bph-38*, *Bph42-2*, and *Bph-45*. The third region, located between markers RM6506 and RM16846, includes *Bph-6*, *Bph12-2*, *Bph-16*, *Bph-18(t)*, *Bph22(t)-2*, *Bph27(t)*, *Bph27*, *Bph-33*, *Bph-34*, and *Bph-44*.

On chromosome 3, several resistance genes have been identified, including *qBph-3*, *Bph11(t)*, *Bph13(t)-2*, *Bph-14*, *Bph14*, *Bph19(t)-1*, and *Bph31*. Similarly, chromosome 6 carries multiple resistance genes, including *Bph3-1*, *Bph4*, *qBph-6*, *Bph20(t)-2*, *Bph25*, *Bph32*, and *Bph-2*. In addition, resistance loci such as *qBph-11*, *qBph-8*, *Bph13(t)-1*, *Bph21(t)-2*, *Bph23(t)-2*, *Bph28(t)*, *Bph37-1*, *Bph38(t)*, and *Bph43* are distributed across chromosomes 1, 2, 8, 10, and 11. These gene clusters may represent closely linked genes, different alleles at the same locus, or the same allele showing variable responses to different brown planthopper biotypes.

### 3.2. Mapping of Genes Resistant to Rice Laodelphax striatellus

*Laodelphax striatellus* is mainly distributed in Japan, China, and South Korea and transmits two major rice diseases, black-streaked dwarf disease and rice stripe disease. To date, only a limited number of genes associated with resistance to the small brown planthopper (SBPH) have been reported in rice. Screening of 25 rice varieties identified three SBPH-resistance QTLs in the rice variety Mudgo: Qsbph2b, Qsbph3d, and Qsbph12, which are located on chromosomes 2, 3, and 12, respectively, and show relatively low contribution rates [26]. The indica rice variety Kasalath also carries SBPH-resistance QTLs, including three QTLs associated with avoidance and two associated with resistance, located on chromosomes 2, 3, 8, and 11. Among these, Qsbph11, mapped between markers S2260 and G257 on chromosome 11, is considered a major resistance locus in Kasalath [27].

In 2013, Wang et al. [28] identified five QTLs for SBPH resistance in the indica rice variety N22: qSBPH2, qSBPH3, qSBPH5, qSBPH7, and qSBPH11, which are located on chromosomes 2, 3, 5, 7, and 11, respectively. Among these, qSBPH7, mapped between markers RM234 and RM429 on chromosome 7 using three different phenotyping methods, was suggested to be the major resistance locus in N22. Subsequently, Zhang et al. [29] identified three additional SBPH-resistance QTLs on chromosomes 3, 7, and 12. Recent genome-wide association studies further confirmed that the gene OsAP47, located on the short arm of chromosome 6, is the first cloned gene conferring resistance to black-streaked dwarf disease [30], indicating that multiple resistance genes may act synergistically. Although these findings provide valuable targets for marker-assisted breeding, QTL expression is influenced by genetic background. Therefore, multi-environment validation and gene pyramid strategies are recommended to improve the stability of resistance [31].

### 3.3. Sogatella Furcifera Resistance Genes

*Sogatella furcifera* Horváth is one of the most destructive piercing–sucking pests in rice-growing regions of Asia. The identification and mapping of resistance genes against this planthopper are essential for breeding insect-resistant rice varieties. To date, nine resistance genes and several quantitative trait loci (QTLs) associated with *S. furcifera* resistance have been reported in rice. The genes *Wbph1* to *Wbph5* were identified using classical genetic approaches. Among these, *Wbph1*, derived from the rice variety N22, is located in the RM13650–RM13478 interval on chromosome 2, while *Wbph2* is linked to the molecular marker RZ667 on the same chromosome, at a distance of approximately 25.6 cM [32]. The gene *Wbph6* is located on the short arm of chromosome 11, about 21.2 cM from the marker RM1667 [33].

The genes *Wbph7* and *Wbph8*, identified from wild rice species, are located at the same chromosomal positions as the brown planthopper resistance genes *Bph14* and *Bph15* [34]. All of these white-backed planthopper resistance genes provide resistance at the seedling stage of rice. Additionally, an egg-killing resistance gene, *Ovc*, derived from the cultivated rice variety Asominori, has been mapped to chromosome 11 [35]. Several other QTLs associated with seedling resistance and egg-killing activity have also been identified [36]. Notably, transgenic lines carrying cloned brown planthopper resistance genes, such as *Bph3*, *Bph14*, and *Bph15*, also demonstrate resistance to white-backed planthoppers. Therefore, rice varieties developed using these genes can offer resistance to both brown and white-backed planthoppers.

### 3.4. Mapping of Orseolia oryzae Resistance Genes

*Orseolia oryzae* is a major pest in rice-producing regions of Asia and Africa. The larvae invade the growing point of rice plants during the seedling and tillering stages, causing the leaf sheaths to form a characteristic gall, commonly known as a “silver shoot”. Affected plants fail to produce panicles, resulting in severe yield losses. Many wild rice species show resistance to this pest, and twelve major resistance genes have been identified, including *Gm1*, *Gm2*, *Gm3*, *Gm4*, *Gm5*, *Gm6*, *Gm7*, *Gm8*, *Gm9*, *Gm10*, *Gm11*, and *Gm12*.

The gene *Gm1* is located within a 0.18 Mb region on chromosome 9, *Gm2* within a 0.66 Mb region on chromosome 4, *Gm4* within a 0.43 Mb region on chromosome 8, and *Gm5* within a narrow 49 kb region on chromosome 12. The genes *Gm6* and *Gm7* are also located on chromosome 4, while *Gm8* and *Gm4* are both found on chromosome 8. The gene *Gm9* spans a region of approximately 0.15–0.25 Mb, and *Gm10* is located on chromosome 9. Both *Gm11* and *Gm5* are present on chromosome 12 but are separated by about 0.28 Mb [37]. The recessive gene *gm12* has been finely mapped to a 0.345 Mb region on chromosome 12. These resistance genes confer protection against different *O. oryzae* biotypes (e.g., biotypes 1, 3, 5, or 1–4) through mechanisms such as hypersensitive response, antibiosis, non-preference, and recessive resistance. Different genes provide resistance to specific biotypes; for example, *Gm1* confers resistance to biotypes 1, 3, and 5, whereas *Gm4* provides resistance to biotypes 1–4 [38]. Using marker-assisted selection (MAS), multiple resistance genes, including *Gm4*, *Gm8*, and *Xa21*, have been successfully introduced into elite rice varieties, resulting in broad-spectrum resistance [39]. Recent studies suggest that *Gm5*, located in the 49 kb region of chromosome 12, is associated with two candidate genes (*Os12g36830* and *Os12g36880*), both of which show higher expression levels in resistant varieties than in susceptible ones [40].

### 3.5. Mapping of Nephotettix cincticeps Resistance Genes in Rice

*Nephotettix cincticeps* is one of the most serious rice pests in tropical and subtropical regions. It not only feeds on rice, causing yield losses, but also carries the Verticillium wilt virus and Tongru virus. Genetic analysis has revealed that fourteen major resistance genes (*Glh1-Glh14*, excluding *Glh10*) have been identified. Among them, *Glh1* (from Pankhari203, located on chromosome 5), *Glh2* (from ASD7, located on chromosome 11), *Glh3* (from IR8, located on chromosome 6), *Glh5* (from ASD8, located on chromosome 8), *Glh6* (from TAPL796, located on chromosome 5), and *Glh14* (from ARC1554, located on chromosome 4) have been the most extensively studied [41]. The green leafhopper resistance genes *Glh1*, *Glh2*, *Glh3*, *Glh4*, *Glh5*, *Glh6*, and *Glh14* are located on chromosomes 5, 11, 6, 3, 8, 5, and 4 of rice varieties Pankhari203, ASD7, IR8, Ptb8, ASD8, TAPL796, and ARC1554, respectively [42]. With the help of classical genetic analysis, molecular marker-assisted selection, and high-throughput sequencing technology, researchers have successfully located these genes to specific chromosomal intervals. For example, *Glh3* was precisely located in the RM587–RM589 region on chromosome 6 [43]. However, to address this challenge, current research is shifting toward multi-gene strategies (such as the *Glh2* + *Glh14* combination) and gene editing technologies (such as CRISPR/Cas9 targeted editing of susceptible genes) to enhance durability and broadness of resistance [44]. Future research should further improve the mapping accuracy of resistance genes and further elucidate the molecular interactions between resistance genes and *N. cincticeps* adaptation, providing a theoretical basis and technical support for the development of durable insect-resistant rice varieties.

### 3.6. Mapping of Genes Resistant to Cnaphalocrocis medinalis

*Cnaphalocrocis medinalis* (Guenée) is found throughout Asia, where its larvae damage rice crops. The larvae curl rice leaves lengthwise, hiding inside and feeding on the epidermis and mesophyll. This damages flowering and fruiting, increases empty shells on rice panicles, and decreases the 1000-grain weight. In China, the rice leaf roller is one of the most serious pests of rice. Although some insect-resistant germplasm resources have been identified in wild and cultivated rice, no major insect-resistant genes have been reported. There are reports that the Chinese variety Chunjiang 6 is resistant to rice leaf roller. Genetic analysis of rice leaf roller resistance using a doubled haploid population of CJ06/TN1 identified several QTLs that improve rice resistance to *C. medinalis*. Although each locus has a small effect, combining multiple QTLs significantly enhances rice resistance to the leaf roller [45].

### 3.7. Rice Stem Borer Resistance Gene Mapping

Compared to the later-developed field of chromosome mapping for stem borer resistance, research on piercing-sucking pests like the brown planthopper has advanced quickly in the past five years. Lei et al. [46] used chromosome segment replacement lines to locate five major QTLs for rice stem borer resistance (*qRSB1*, *qRSB3*, *qRSB4*, *qRSB6*, *qRSB10*) on chromosomes 1, 3, 4, 6, and 10, and developed tightly linked markers such as CS0138, CS0333, R4M43, CS0610, and CS1002, providing important chromosome resources for molecular marker-assisted breeding of rice resistant to rice stem borer.

**Figure 2:**
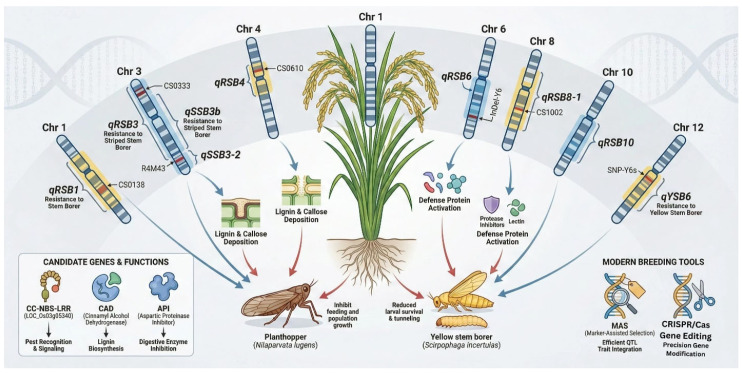
Rice stem borer-resistant gene mapping. Blue arrows show resistance genes and signaling pathways, while red arrows show plant defense responses against stem borers.

Building on this foundation, both domestic and international studies have further broadened the range of resistance loci. A major QTL for rice stem borer resistance, qSSB3b (RM7–RM16; LOD = 8.7; contribution rate 21.4%), was identified in the Nantong 11 × TN1 F_2_:_3_ population. Together, they pinpointed the CC-NBS-LRR candidate gene, LOC_Os03g05340, within a 34 kb region. For the yellow stem borer (*Scirpophaga incertulas*), Gokulan et al. [47] used a four-year multi-site inoculation combined with a 6K SNP array to precisely map the major locus qYSB6 to 2.34–2.41 Mb on the short arm of chromosome 6 (LOD = 11.2, contribution rate 24.1%). They developed functional markers, InDel-Y6 and SNP-Y6s, which showed a highly significant negative correlation (r = –0.83) with lignin content and field *S. incertulas* rate. These markers were then used to create a *qYSB6* + *Bph14* + *Pi9* three-gene polymorphism in the Huajing 0748 rice background, enabling simultaneous improvement of resistance to stem borer, planthopper, and rice blast [48]. However, stable QTL-marker-phenotype associations have been established on chromosomes 1, 3, 4, 6, 8, 10, and 12, providing a reliable chromosomal resource for map-based cloning, molecular breeding, and multigene rotation. Wani et al. [49] also noted in their review that the loci qRSB1, qSSB3b, qSB8-1, and qYSB6 recur across diverse genetic backgrounds, with effect sizes ranging from 15–24%, and colocalize with genes encoding lignin, callose, and defense proteinase inhibitors (Figure 2). The article stresses the importance of implementing MAS using tightly linked SNP/InDel markers and combining these with gene editing tools like CRISPR/Cas for functional validation of candidate genes such as CC-NBS-LRR, CAD, and API to speed up the development of broad-spectrum, durable stem borer-resistant varieties.

## 4. Insect-Resistant Genes

Cloning and identifying insect-resistant genes are essential for breeding long-lasting, broad-spectrum insect-resistant rice. Over the past decade, Chinese teams first isolated Bph14 through map-based cloning [50]. Then, they systematically analyzed the differential resistance of *Bph9* polyallelic lines (*Bph1/2/7/9/10/18/21/26*) within the 12 L 19.1–24.4 Mb region to various biotypes [51,52]. They also showed that the membrane-localized LecRK cluster *Bph3* [26] and the single gene *Bph15* [52] activate Pattern-Triggered Immunity (PTI) by sensing β-glucan in brown planthopper salivary fluid. Conversely, intracellular CC-NB-LRR or CC-NB proteins (Bph14 and Bph9) and the exosomal LRR-only protein *Bph6* also trigger PTI [53]. These receptors detect effectors that initiate Effector-Triggered Immunity (ETI), which collectively upregulate callose and lignin deposition to confer broad-spectrum resistance. The nuclear-localized B3 protein bph29 [54] and the SCR protein Bph32 [35] further support resistance through SA/JA signaling crosstalk and an unknown membrane mechanism, respectively (Supplementary Table S2).

The cloned rice insect resistance genes identified so far mainly function through endogenous defense pathways, including the detection of insect feeding signals, activation of pattern-triggered and effector-triggered immunity, and downstream signaling steps such as calcium signaling, MAPK cascades, and salicylic acid–jasmonic acid cross-communication, which collectively reduce insect feeding and damage [50,51,52,53,54,55].

Although several insect-resistance genes have been identified in rice, the mechanisms by which rice detects insect invasion and interacts with insects remain unclear. Functional genomic research on rice insect resistance is still in its early stages [56]. Numerous molecular biology techniques, such as suppression subtractive hybridization, gene chips, transcriptomics, proteomics, and metabolomics, have been used to study rice resistance to brown planthoppers and rice stem borers [57,58]. These techniques have accumulated a wealth of data, offering an increasingly clear understanding of the mechanisms behind insect resistance.

When insects feed on rice, the plant cells recognize specific elicitors or effectors in insect saliva through pattern recognition receptors on the cell surface. For example, the rice brown planthopper resistance gene *Bph3* may function as a pattern recognition receptor. This recognition quickly triggers early signaling events (Ca^2+^ influx/oscillations, ROS bursts, and MAPK cascade activation), which then activate downstream phytohormone signaling (including JA/SA) and regulate defense genes [59].

### 4.1. Rice Recognition of Insect Feeding Signals

Rice plants detect insect feeding by recognizing insect-derived elicitors and effectors in saliva, which are perceived by membrane-bound pattern recognition receptors or intracellular immune receptors, triggering defense signaling. The process by which rice identifies insect feeding signals involves a highly conserved and layered molecular defense network. To date, two main types of rice insect resistance genes have been cloned: the cell membrane receptor kinase LecRK and the intracellular NBS-LRR. In rice, responses to pests like the brown planthopper (BPH) involve two defense systems: Lectin Receptor Kinase (LecRK) and Nucleotide-Binding Site Leucine-Rich Repeat (NBS-LRR), which detect molecular patterns (Host-Associated Molecular Patterns, HAMPs) and effector signals released during insect feeding, respectively, leading to PTI and Effector-Triggered Immunity (ETI) responses [60]. Studies show that when insects feed on crops, they secrete saliva into the plant. Substances produced by these secretions or by degradation or modification of plant cell components can act as HAMPs, DAMPs, or effectors (such as PAMPs/MAMPs), triggering plant resistance responses. Various elicitors and effectors have been identified in insects feeding on other crops, such as β-glucosidase in the oral cavity of *Pieris rapae* larvae, which can induce the release of volatile compounds from cabbage leaves [61], and Caeliferin A and B in locust oral secretions, which stimulate the release of volatile terpenes from maize [62]. The effector gene C002, identified through transcriptome analysis of pea aphid salivary glands (RNAi inhibition of its expression can reduce aphid survival and reproduction rates) [63], and the effector gene vH13 in the ryegrass midge, can overcome the wheat resistance gene H13 [64].

However, the study of clear elicitors and effectors in insects feeding on rice remains limited. Exploratory studies have been conducted on the effector proteins and virulence genes of the brown planthopper (*N. lugens*). For example, the salivary sheath protein (NlShp) has been isolated from the brown planthopper [64]. This protein is a component of the salivary sheath, and dsRNA inhibits its expression, affecting the brown planthopper’s survival [43]. Furthermore, the recessive virulence gene *vBph1*, which overcomes rice resistance conferred by *Bph1*, has been identified [65]. The accumulation of whole-genome sequencing data for the brown planthopper, along with salivary gland transcriptome and proteome analysis, will aid in identifying its effector proteins and systematically analyzing virulence factors. When pests such as brown planthoppers, white-backed planthoppers, and rice borers feed, the effector proteins in their saliva (such as NlShp of brown planthoppers and β-glucosidase in the oral secretions of rice borers) are recognized as HAMPs by rice cells [66,67]. Rice uses LecRKs (such as Bph3 and Bph15) as PRRs to recognize salivary glycoproteins and activate PTI; intracellular NLR proteins (such as Bph14 and Bph9) identify effectors (such as BISP) to trigger ETI [52,68]. This recognition process rapidly activates intracellular Ca^2+^ oscillations, NADPH oxidase-mediated ROS bursts, and MAPK cascade activation (such as OsMPK3/6) [69,70,71]. Downstream signals activate callose synthase through the Ca^2+^-CDPK module, leading to blockage of the vascular sieve plate; ROS and hormone signals work together to regulate the synthesis of insect-resistant compounds [72].

**Figure 3:**
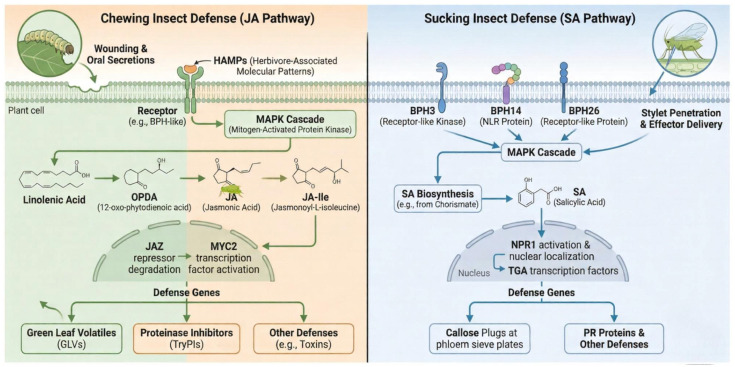
Molecular mechanisms of insect resistance in rice. When insects feed on rice, insect-associated molecular patterns (HAMPs) or damage-associated molecular patterns (DAMPs) can be sensed by rice pattern recognition receptors (PRRs) (such as the brown planthopper resistance gene *Bph3*). Simultaneously, Ca^2+^ influx activates the MAPK cascade, leading to pattern-triggered immunity (PTI). Insect-secreted effectors can prevent PTI. Cloned rice brown planthopper resistance genes, such as *Bph14* and *Bph26*, can sense effectors, while *Bph29* may sense effectors through other proteins, activating effector-triggered immunity (ETI). Insect-resistant genes bind to transcription factors, activating the salicylic acid (SA) signaling pathway. Expression of salicylic acid-responsive defense genes and callose deposition enhances rice resistance to brown planthoppers. When chewing insects (such as rice stem borer) feed on rice, the MAPK cascade activates JA synthesis and signaling pathways, resulting in the production of TryPIs, H_2_O_2_, and defense genes that activate the JA response, thus inhibiting the rice stem borer’s feeding and growth. Additionally, green leaf volatiles (GLVs) primarily provide indirect defense by repelling insect feeding and attracting natural enemies of insects.

Rice coordinates its defense through hormone reprogramming: the SA pathway mainly responds to piercing–sucking pests such as planthoppers; the JA and ET pathways respond more to chewing pests like stem borers; and growth-related hormones such as GA are antagonistically downregulated to redirect resources toward defense (Figure 3) [70,71]. Additionally, volatile compounds released by rice, such as (E)-β-caryophyllene, can indirectly attract natural enemies, creating a triple nutritional defense [73]. This integrated insect resistance mechanism, which includes “recognition–signal transduction–physiological response–indirect defense”, provides a theoretical foundation for developing multi-resistant rice varieties.

In addition to pattern-triggered immunity, rice insect resistance also involves effector-triggered immunity (ETI), which is activated through specific interactions between resistance (R) genes and insect-derived avirulence effectors. Several cloned rice R genes, such as *Bph14*, *Bph6*, *Bph9*, and *Bph29*, encode intracellular immune receptors that perceive planthopper salivary effectors and initiate ETI responses. For example, the brown planthopper salivary effector BISP directly interacts with *Bph14*, leading to activation of downstream immune signaling, callose deposition, and restriction of insect feeding [67,74]. These R gene–effector interactions highlight the importance of ETI in rice–insect coevolution and provide a mechanistic basis for durable and broad-spectrum insect resistance.

### 4.2. Transmission of Insect Resistance Signals in Rice

When rice plants detect insect feeding signals, they quickly activate a complex intracellular signaling network mainly composed of calcium (Ca^2+^) signals, reactive oxygen species (ROS), mitogen-activated protein kinase (MAPK) cascades, phytohormones, and multiple transcription factors (TFs), which trigger specific defense responses against insect attacks.

#### 4.2.1. Calcium Signaling

Calcium ion (Ca^2+^) is a universal second messenger in plant cells and plays a central role in converting external stimuli into intracellular physiological responses [75]. Insect feeding can quickly trigger the influx of extracellular Ca^2+^, leading to oscillations in cytoplasmic Ca^2+^ concentrations [76,77]. This change in Ca^2+^ signaling is crucial for initiating downstream defense events. In defenses triggered by brown planthopper feeding or its salivary proteins (such as NlSEF1), Ca^2+^ influx is one of the early responses that activate subsequent reactions (such as reactive oxygen species burst and callose deposition) [73]. Studies have shown that Ca^2+^ signaling directly regulates the activation of respiratory burst oxidase homologs (RBOHs) to produce ROS and may activate specific protein kinases through sensors like calmodulin [75,76]. More importantly, Ca^2+^ serves as a key cofactor of callose synthase. Activation of Ca^2+^ signaling can directly prompt rapid callose deposition in sieve tubes, forming a physical barrier that effectively hinders feeding by piercing–sucking pests such as brown planthoppers [78,79]. Therefore, Ca^2+^ signaling acts as a central link connecting early recognition and later execution of defense.

#### 4.2.2. Reactive Oxygen Species (ROS)

Plants face various types of biotic stress, such as insect feeding, which produces ROS that can participate in defense signaling pathways and trigger a hypersensitive response (HR) at the feeding site [80]. When brown planthoppers feed on OsHI-LOX-inhibited transgenic plants, cell death similar to the hypersensitive response occurs in the outermost leaf sheath of the transgenic plants [81]. While localized cell death can inhibit pathogen growth and spread, this effect is clearly ineffective against mobile insects like brown planthoppers. Reducing the JA content in rice leads to a decrease in H_2_O_2_, which in turn promotes feeding by rice stem borer and rice leaf roller [82]. Therefore, H_2_O_2_ is likely a byproduct of activating the JA signaling pathway.

#### 4.2.3. MAPK Cascade

MAPK cascades are highly conserved signaling modules in eukaryotes, serving as a bridge connecting upstream perception and downstream defense gene expression in plant innate immunity [83]. Studies have demonstrated that the MAPK cascade is a crucial downstream pathway for multiple brown planthopper resistance genes involved in mediating defense responses [84]. MAPK influences insect resistance by regulating the signaling pathways of key defense hormones (SA, JA, and ET). For instance, inhibiting the expression of OsMPK3 decreases JA levels, weakens the JA signaling pathway, and reduces TrypPI accumulation, leading to decreased rice resistance to brown planthoppers [85]. Additionally, *OsMPK3* and *OsMPK6* can be activated by upstream LRR-RLK receptor kinases (such as OsLRR-RLK1) and positively regulate the synthesis of JA and ET, thereby strengthening resistance to rice stem borer [86]. Conversely, the MAPK cascade can also enhance resistance through pathways independent of core hormones. In resistance mediated by the gene *Bphi008a*, the *OsMKKK18/24-OsMKK4-OsMPK3/6* module is activated [82,87]. Furthermore, during resistance to brown planthoppers, *OsMPK5/12* can directly phosphorylate and activate transcription factors OsERF1 and OsEREBP1, significantly increasing their expression levels and ultimately boosting rice resistance to brown planthoppers [88]. These findings suggest that the MAPK cascade interacts crosswise with multiple resistance pathways, forming a complex signaling network that finely regulates rice defenses.

#### 4.2.4. Phytohormones

Phytohormones are key signaling molecules that control insect resistance in rice. Their response patterns heavily depend on the insect feeding mode (piercing–sucking versus chewing) and the genetic background of the rice variety. The interaction between rice and brown planthoppers resembles plant–pathogen interactions. After brown planthopper feeding, rice plants exhibit significant changes in the transcriptome, proteome, and metabolome. Genes involved in plant defense and macromolecule degradation are upregulated, while genes related to photosynthesis and cell growth are downregulated [54,89]. In resistant rice varieties, defense against brown planthoppers mainly involves activation of salicylic acid (SA)-dependent signaling pathways. When resistant rice plants carrying the brown planthopper resistance genes *Bph14* and *Bph29* are attacked, the expression of SA biosynthesis-related genes (*EDS1*, *PAD*, *PAL*, and *ICS1*) and SA levels increase. Conversely, jasmonic acid (JA) levels and the expression of genes involved in JA and ethylene signaling (*LOX*, *AOS2*, and *EIN2*) are significantly lower than in susceptible rice plants [51,54]. Similar defense responses have been reported in other studies of rice reactions to brown planthopper feeding [29].

*NPR1* and *WRKY45* are key regulators of the SA signaling pathway [90]. Feeding by planthoppers significantly induces *NPR1* expression in resistant rice plants [29,91]. However, suppression of *WRKY45* expression reduces planthopper settling, feeding, and survival on rice plants [92], suggesting that *NPR1* positively regulates rice resistance to planthoppers, whereas *WRKY45* may play a negative regulatory role. Other studies have shown that increased expression and activity of the JA biosynthesis gene *LOX1* enhance rice resistance to brown planthoppers, while reduced *LOX1* expression weakens resistance [89,93]. These findings indicate that different signaling pathways are activated in resistant and susceptible rice plants following planthopper feeding. In addition, the hypersensitive response (HR) is an important component of effector-triggered immunity and is closely linked to SA signaling. HR is characterized by rapid, localized cell death at feeding sites, which restricts insect feeding and limits the spread of herbivore-associated pathogens, thereby contributing to overall resistance against brown planthoppers.

The JA signaling pathway is crucial in plant defense against chewing insects. In rice plants, the transcription of genes related to the JA signaling pathway is activated after feeding by *Chilo suppressalis* [70,94]. In rice, *OsPLDα*, *OsLOX*, *OsAOS*, and *OsCOI1* are genes involved in the JA pathway, playing roles in JA synthesis and signal transduction [95]. Inhibiting these genes in rice can lower levels of JA, trypsin inhibitors (TryPIs), H_2_O_2_, and volatiles, which in turn promotes the feeding and growth of *C. suppressalis* and rice leaf roller larvae. However, reducing the expression of these genes does not affect brown planthoppers or decrease their feeding and survival rates [94,95]. This indicates that JA has contrasting roles in rice defense against chewing insects versus piercing–sucking insects.

## 5. Physiological Mechanisms of Rice Insect Resistance

Through long-term coevolution with insects, plants have developed a variety of defense strategies to protect themselves. From a physiological perspective, plant resistance to insects is usually categorized into three mechanisms: antixenosis, antibiosis, and tolerance [95]. Antixenosis refers to plant traits that discourage insects from settling, feeding, or laying eggs during host selection. Antibiosis occurs when insects can feed or oviposit on a plant, but plant defenses negatively impact insect survival, feeding behavior, growth, or reproduction, ultimately leading to higher mortality. Tolerance describes the plant’s ability to withstand insect damage and continue growing and surviving despite tissue injury. Tolerance is a plant’s response to insect feeding, with the primary mechanism being compensation. Rice plants, especially those with many tillers, can compensate for damage caused by insect feeding. Studies have shown that even when rice water flies damage 75% of rice seedlings, it does not result in significant yield loss [96]. However, this compensatory ability decreases as the plant matures. Indicators of compensation include reduced plant weight, yellowing leaves, and lower yields [97]. Currently, the genetic mechanisms underlying tolerance remain unclear.

Repellency and antibiotic resistance are the insects’ responses to plants. The molecular mechanisms behind these processes are not fully understood. Some secondary metabolites, volatiles, and defense proteins—such as protease inhibitors, lectins, or specific enzymes—can make plants less attractive to insects or hinder their growth, development, survival, and egg-laying. Brown planthoppers use their mouthparts to pierce epidermal cells, penetrate the cell wall, secrete saliva into the cells, and digest phloem sap. After feeding on plants, they may encounter chemicals produced by the plants, which are detected by the planthoppers’ mouthparts and can cause repellency or influence their feeding behavior. These chemicals may originate from alkyl and carbonyl compounds in the epidermal wax layer. For example, the proportion of short carbon chains in the epidermal wax layer of insect-resistant rice is higher [98].

Mechanical barriers can prevent insects from feeding on the phloem. In insect-resistant rice, brown planthoppers spend significantly less time feeding on the phloem compared to susceptible rice. In the highly resistant rice variety B5, brown planthoppers spend only a quarter of their time feeding on the phloem, unlike in susceptible rice [99,100]. In resistant plants, the presence of antifeedants and the lack of phagostimulants can also reduce brown planthopper feeding. Asparagine, considered an attractant, is found at lower levels in the resistant rice Mudgo. Soluble silicic acid, oxalic acid, and sterols in resistant plants are regarded as antifeedants [101,102]. Water-soluble benzoates exhibit ovicidal activity against white-backed planthoppers at concentrations of 6.4 ppm [103]. A well-known plant defense compound is trypsin inhibitor (PI), which plays a key role in plant defense against insect feeding. In resistant rice, the PI gene is upregulated by brown planthopper feeding [50], and PI accumulation is also triggered by rice stem borer feeding [104,105].

For rice pests with chewing mouthparts, such as *C. suppressalis*, the plant’s morphology, structure, and physiological and biochemical factors may all contribute to resistance against insects [57]. Dense leaf sheaths can prevent newly hatched *C. suppressalis* larvae from feeding on the inner part of the leaf sheath [106]. P-methyl acetophenone can attract moths and *C. suppressalis* to lay eggs [107,108], while allomone can inhibit *C. suppressalis* from laying eggs and prevent egg hatching, larval growth, and survival [109,110]. Additionally, the silicon content in rice can increase resistance to *C. suppressalis*. Feeding on rice plants with high silicon levels can quickly cause the mandibles of *C. suppressalis* larvae to wear out, reducing their food intake and leading to death [111,112,113].

Rice volatiles include many important secondary metabolites that mainly serve as signals to attract parasitic wasps and natural enemies of herbivorous insects, thus providing indirect plant defense [114,115]. Feeding by brown planthoppers and rice stem borers activates the production of organic volatiles in rice, thereby attracting the parasitic wasps of rice leafhoppers. In 2012, Xiao et al. [116] reported that linalool (S-linalool), induced by brown planthopper feeding in rice, can attract parasitic wasps and natural enemies of brown planthoppers. Green leaf volatiles (GLVs), a branch of the lipoxygenase pathway, play an important role in plant defense against planthopper feeding. Rice OsHPL3 catalyzes the production of GLVs, which enhances rice resistance to brown planthoppers and attracts parasitic wasps of rice leafhoppers [117].

## 6. Cultivation of Insect-Resistant Rice

Planting insect-resistant rice varieties is an effective and environmentally friendly way to control pests. Zhang et al. [10] introduced the idea of Green Super Rice breeding, focusing on developing rice varieties resistant to multiple pests. Traditional insect-resistant rice breeding depends on screening for resistant traits, which involves growing large breeding populations and evaluating agronomic traits, yield, resistance, and tolerance. Using this method, the International Rice Research Institute has created many rice lines resistant to brown planthoppers, leafhoppers, and rice stem borers [110]. However, developing populations and testing resistance are time-consuming and labor-intensive tasks, and close cooperation between breeders and entomologists is crucial for success [118,119].

The use of molecular markers has significantly advanced the breeding of insect-resistant rice. Molecular marker-assisted selection provides several advantages and has been extensively used in rice breeding. Using this method, many rice lines resistant to brown planthoppers, white-backed planthoppers, and rice gall midges have been successfully created. With the frequent outbreaks of brown planthoppers, more focus has been placed on developing durable resistance through stacking multiple resistance genes. Rice varieties with two resistance genes generally demonstrate stronger pest resistance and avoidance than those with a single gene [120,121]. Compared to traditional hybrid rice, hybrids with one resistance gene show improved pest resistance, while hybrids with two or more genes show even greater resistance [122].

In China, many research institutions have developed brown planthopper-resistant restorer lines, sterile lines, and hybrid rice combinations, some of which have been officially released and widely adopted in production. For example, the research team led by Academician Zhu Yingguo at Wuhan University developed the brown planthopper-resistant photo-thermosensitive genic male sterile line *Bph68S* and the Honglian-type cytoplasmic male sterile line Luohong 4A, both carrying the *Bph14* and *Bph15* genes [123]. They also developed the two-line hybrid rice Liangyou 234, which has been approved for cultivation in Hubei and Anhui Provinces. Additionally, Nanjing Agricultural University completed the map-based cloning of the *Bph3* gene and incorporated this resistance gene cluster into the susceptible rice variety Ningjing 3. The resulting lines show high resistance to brown planthoppers at both seedling and mature stages. Moreover, these lines also demonstrate strong resistance to white-backed planthoppers, providing a foundation for breeding rice varieties resistant to multiple planthopper species [124].

In recent years, advancements in biotechnology have opened new pathways for pest control. Transgenic rice varieties can be modified to enhance insect resistance by inserting insecticidal genes, such as the *B. thuringiensis* endotoxin gene (Bt) and the snowdrop agglutinin gene (GNA). Currently, no germplasm resources exhibit high resistance to rice stem borer. At the same time, the Bt gene is highly effective against lepidopteran insects and is the preferred choice for controlling rice stem borer and rice leaf roller [125]. GNA is another important insecticidal gene that targets Hemipteran insects with piercing–sucking mouthparts but does not possess the toxicity of Bt. Transgenic rice expressing lectins can slow the growth, development, and reproduction of insect pests [126,127]. However, lectins are not as effective against planthoppers and leafhoppers as Bt is against lepidopteran insects and rice insect-resistant genes.

## 7. Future Directions

Future research on rice insect resistance will face multiple challenges and opportunities. First, cloned resistance genes mainly target a few pests, such as the brown planthopper. Resistance genes targeting other major pests, like rice leaf roller and the black planthopper, are still rare, requiring ongoing discovery of new resistance genes from wild rice species and local varieties. Second, pest populations show rapid genetic variation and strong adaptive evolution, and widespread, long-term use of a single resistance gene can easily lead to resistance breakthroughs. Therefore, efforts should focus on strengthening multi-gene stacking, rotational strategies, and developing broad-spectrum resistant varieties [128]. To reduce the overuse of insecticides and lower the risk of resistance, future pest management should shift from chemical control to sustainable, eco-friendly methods, emphasizing the development of broad-spectrum, multi-resistant pest control options through molecular breeding, functional genomics, and ecological regulation [129]. Additionally, although some resistance genes have been cloned, the molecular mechanisms behind rice’s recognition of insect feeding signals, the functional roles of insect saliva effectors, and the plant immune regulatory network remain largely unknown. Future work will need to combine multi-omics, gene editing, and artificial intelligence technologies to systematically uncover the molecular basis of crop–pest interactions and coevolution. Furthermore, climate change may impact pest occurrence and distribution, increasing the demand for resistant varieties that can adapt and remain stable. As a result, developing a climate scenario-based resistance evaluation and prediction system is urgently needed. Looking ahead, research on rice insect resistance should shift from single-gene resistance to systemic resistance, promote resistance gene diversification, deepen our understanding of mechanisms, and advance intelligent breeding technology. By integrating functional genomics, molecular design breeding, and ecological regulation strategies, we can develop long-lasting, broad-spectrum, multi-resistant super rice varieties, supporting global food security and sustainable agriculture.

## 8. Summary

Rice, the staple food for half the world’s population, experiences an annual loss of 28–34% due to insect pests. The limitations of chemical pesticides make breeding resistant varieties a key strategy for sustainable pest management. This article reviews the latest advances in molecular breeding of insect-resistant rice. Key resistance genes such as *Bph14*, *Bph3*, and *Bph29* have been cloned from both cultivated and wild rice species using functional genomics. A two-layered immune mechanism has been identified in rice, involving the recognition of insect feeding signals by membrane receptor kinases and intracellular NLR proteins, which activate the SA/JA hormone pathways, MAPK cascade, calcium signaling, and bursts of reactive oxygen species. Multi-resistant varieties have been developed using molecular marker-assisted selection and multi-gene stacking technology. To tackle challenges like pest adaptation and climate change, future research should explore wild rice resources, investigate interactions between insect effector proteins and plant immunity, and incorporate multi-omics, gene editing, and molecular design breeding to develop durable, broad-spectrum insect-resistant green super rice.

## Data Availability

No new data were created or analyzed in this study.

## Supplementary Materials

The following supporting information can be downloaded at: https://www.mdpi.com/article/10.3390/insects17010111/s1, Table S1: BPH-resistant genes/QTLs in rice.
