# Supplementary material for: Molecular Mechanisms of Insect Resistance in Rice and Their Application in Sustainable Pest Management
Source: Insects. 2026 Jan 19;17(1):111. doi: 10.3390/insects17010111 (PMC12842263; doi:10.3390/insects17010111)
Supplement: Supplementary file 1 [file insects-17-00111-s001.zip › insects-4058669-supplementary.pdf]

Table S1. BPH-resistant genes/QTLs in rice.

Gene	Germplasm	Chr.	Encoded protein	Subcellular localization	Expression pattern	Resistance against	References
BPH1	Mudgo	12L	CC-NB-NB-LRR	Endomembrane system	Vascular bundle	BPH	Zhao et al. 2016
bph2	ASD7	12L	CC-NB-NB-LRR	Endomembrane system	Vascular bundle	BPH	Tamura et al. 2014
BPH3	Rathu Heenati	4S	Lectin receptor kinases	Plasma membrane	Vascular bundle	BPH and WBPH	Liu et al. 2015
BPH6	Swarnalata	4L	Atypical LRR	Exocyst	Vascular bundle/sclerenchyma	BPH and WBPH	Guo et al. 2018
bph7	T12	12L	CC-NB-NB-LRR	Endomembrane system	Vascular bundle	BPH	Du et al. 2009
BPH9	Pokkali	12L	CC-NB-NB-LRR	Endomembrane system	Vascular bundle	BPH	Zhao et al. 2016
BPH10	IR65482–4–136-2-2	12L	CC-NB-NB-LRR	Endomembrane system	Vascular bundle	BPH	Zhao et al. 2016
BPH14	B5	3L	CC-NB-LRR	Nucleus and cytoplasm	Vascular bundle	BPH	Du et al. 2009
BPH15	B5	4S	Lectin receptor kinase	Plasma membrane	Vascular bundle	BPH	Cheng et al. 2013
BPH18	IR65482-7-216-1-2(*O. australiensis*)	12L	CC-NB-NB-LRR	Endomembrane system	Vascular bundle	BPH	Ji et al. 2016
BPH21	IR71033-121-15 (*O. minuta*)	12L	CC-NB-NB-LRR	Endomembrane system	Vascular bundle	BPH	Zhao et al. 2016
BPH26	ADR52	6S	CC-NB-NB-LRR	Endomembrane system	Vascular bundle	BPH	Zhang et al. 2024
bph29	RBPH54	6S	B3 DNA-binding domain	Nucleus	Vascular bundle	BPH	Zhang et al. 2015

Bph30	AC-1613	4S	LRD	Endomembrane system	Vascular bundle	BPH	Shi et al. 2021
BPH32	PTB33	6S	Unknown SCR domain	Plasma membrane	Vascular bundle	BPH	Ren et al. 2016
Bph37	SE382	6L	CC-NB	-	Vascular bundle	BPH	Zhou et al. 2021
Bph40	SE232, SE67, C334	4S	LRD	-	Vascular bundle	BPH	Shi et al. 2021

Table S2. The cloned insect-resistant genes in rice

**Genes/QTLs**	**Chromosome**	**Germplasm**	**Linked Markers**	**Position (Mbp)**	**PEV**	**Reference**
Bph1	12L	Mudgo	pBPH4-pBPH14	22.86	Major gene	Cha et al. 2008
Bph2 (bph2)	12L	ASD7	RM463-RM7102	22.87~22.89	Major gene	Sun et al. 2006
Bph3-1 (Bph3) #	6S	Ptb33, Rathu Heenati	RM589-RM588	1.38~1.48	Major gene	Jairin et al. 2007
Bph3-2 (Bph3) *#	4S	Rathu Heenatis	RHD9-RHC10	6.20~6.97	Major gene	Liu et al. 2015
Bph4 (bph4)	6S	Babawee RM589-	RM586	1.38~1.47	58.8–70.1%	Jairin et al. 2010
Bph5 (bph5)	—	ARC10550	—	—	Major gene	Khush et al. 1985
*Bph6**	4L	Swarnalata	H-Y9	21.40	Major gene	Guo et al. 2018
Bph7	12L	T12	RM3448-RM313	19.95~20.87	38.3%	Qiu et al. 2014
Bph8 (bph8)	—	Chin Saba	—	—	Major gene	Nemoto et al. 1989
*Bph9**	12L	Pokkali	InD2-RsaI	22.85~22.97	Major gene	Wang et al. 2021
Bph10 (bph10)	12L	*O. australiensis*	RG457	19.55~26.98	Major gene	Ishii et al. 1994
Bph11(t) (bph11)	3L	*O. officinalis*	G1318	35.60~35.80	Major gene	Renganayaki et al. 2002

Bph12-1 (Bph12) #	4S	B14 (*O. officinalis*)	RM16459-RM1305	5.21~5.56	73.8%	Qiu et al. 2012
Bph12-2 (bph12) #	4L	*O. officinalis*	G271-R93	20.34~21.31	Major gene	Hirabayashi et al. 1998
Bph12(t)	4S	*O. latifolia*	RM261-RM8213	4.44~6.57	70.6 %	Yang et al. 2002
Bph13(t)-1 [Bph13(t)] #	2L	*O. eichingeri*	RM240-RM250	31.50~32.78	90%	Liu et al. 2001
Bph13(t)-2 [Bph13(t)] #	3S	IR54745-2-21 (*O. officinalis*)	RG100-RG191	5.18~5.70	Major gene	Renganayaki et al, 2002
Bph14 *	3L	B5 (*O. officinalis*)	SM1-G1318	35.68~35.70	Major gene	Du et al. 2009
Bph15 *	4S	B5 (*O. officinalis*)	RG1-RG2	6.68~6.90	Major gene	Yang et al. 2002
Bph16	4L	*O. officinalis*	G271-R93	20.17~21.14	Major gene	Hirabayashi et al. 1998
Bph17	4S	Rathu Heenati	RM8213-RM5953	4.44~9.38	Major gene	Sun et al. 2005
Bph18(t) [bph18(t)]	4L	*O. rufipogon*	RM273-RM6506	24.05~25.05	Major gene	Rongbai et al. 2010
Bph18 *	12L	IR65482-7-216-1-2 (*O. australiensis*)	BIM3-BN162	22.88	Major gene	Ji et al. 2016
Bph19(t)-1 [bph19(t)] #	3S	AS20-1	RM6308-RM3134	7.18~7.24	Major gene	Chen et al. 2006
Bph19(t)-2 [bph19(t)] #	12L	*O. rufipogon*	RM17	26.98	Major gene	Rongbai et al. 2010
Bph20(t)-1 [Bph20(t)] #	4S	IR71033-121-15 (*O. minuta*)	B42-B44	8.76	Major gene	Rahman et al. 2009
Bph20(t)-2 [bph20(t)] #	6S	*O. rufipogon*	BYL7-BYL8	0.47~0.53	Major gene	Yang et al. 2012
Bph21(t)-1 [Bph21(t)] #	12L	IR71033-121-15 (*O. minuta*)	S12094A-B122	24.20~24.36	Major gene	Rahman et al. 2009
Bph21(t)-2 [bph21(t)] #	10S	*O. rufipogon*	RM222-RM244	2.62~5.00	Major gene	Yang et al. 2012

Bph22(t)-1[Bph22(t)] #	4L	*O. glaberrima*	RM471-RM5742	18.99~21.56	Major gene	Ram et al. 2010
Bph23(t)-1 [Bph23(t)] #	—	*O. minuta*	—	—	Major gene	Deen et al. 2010
Bph22(t)-2 [bph22(t)] #	4L	*O. rufipogon*	RM8212-RM261	19.11~19.57	11.3%	Hou et al. 2011
Bph23(t)-2 [bph23(t)] #	8L	*O. rufipogon*	RM2655-RM3572	16.63~17.07	14.9%	Hou et al. 2011
Bph24(t) [bph24(t)]	—	IR73678-6-9-B (*O. rufipogon*)	—	—	Major gene	Deen et al. 2010
Bph25	6S	ADR52	S00310-RM8101	0.21	Major gene	Myint et al. 2012
*Bph26**	12L	ADR52	DS72B-DS173B	22.87~22.89	Major gene	Tamura et al. 2014
Bph27	4L	GX2183 (*O. rufipogon*)	RM16846-RM16888	19.12~19.50	Major gene	Huang et al. 2013
Bph27(t)	4L	Balamawee	Q52-Q20	20.79~21.33	Major gene	He et al. 2013
Bph28(t)	11L	DV85	Indel55-Indel66	16.90~16.96	Major gene	Wu et al. 2014
*Bph29 (bph29)**	6S	RBPH54 (*O. rufipogon*)	BYL8-BID2	0.48~0.49	Major gene	Wang et al. 2015
*Bph30**	4S	AC-1613	SSR28-SSR69	0.92~0.94	Major gene	Shi et al. 2021
Bph31	3L	CR2711-76	PA25-RM2334	26.25~26.57	Major gene	Prahalada et al. 2017
*Bph32**	6S	Ptb33	RM19291-RM8072	12.23~12.36	Major gene	Wang et al. 2015
Bph33	4S	KOLAYAL, PPLIYAL	H99-H101	19.29~19.79	Major gene	Hu et al. 2018
Bph34	4L	IRGC104646 (*O. nivara*)	RM17007-RM1699	21.32~21.47	Major gene	Kumar et al. 2018
Bph35	4S	RBPH660 (*O. rufipogon*)	RM3471-PSM20	6.28~6.94	51.27%	Zhang et al. 2020
Bph36	4S	GX2183 (*O. rufipogon*)	S13-X48	6.46~6.50	Major gene	Li et al. 2019
Bph37-1 (Bph37) #	1L	IR64	RM302-YM35	19.10~19.20	36.9%	Yang et al. 2019

Bph37-2 (Bph37) *#	6S	SE382	—	3.45	Major gene	Zhou et al. 2021
Bph38(t)	1L	Khazar	SNP693369-id 10112165	20.80~20.90	35.91%	Balachiranjeevi et al. 2019
Bph38	4L	GX2183 (*O. rufipogon*)	YM112-YM190	15.00~15.10	Major gene	Yang et al. 2020
*Bph40**	4S	SE232, SE67, C334,	—	4.48~4.49	Major gene	Shi et al. 2021
Bph41-1 (Bph41) #	4S	SWD10	SWRm_01617-SWRm_01522	0.90~1.10	Major gene	Tan et al. 2022
Bph41-2 (Bph41) #	4S	GXU202 (*O. rufipogon*)	W4_4_3-W1_6_3	4.68~4.78	Major gene	Wang et al. 2022
Bph42-1 (Bph42) #	4S	SWD10	SWRm_01695-SWRm_00328	20.60~21.80	Major gene	Tan et al. 2022
Bph42-2 (bph42) #	4S	*O. rufipogon*	RM16282-RM16335	9.07~9.58	29%	Kaur et al. 2022
Bph43	11S	IRGC 8678	InDel16_22-InDel16-30 1	6.79~16.90	Major gene	Kim et al. 2022
Bph44	4L	Balamawee	Q31-RM17007	21.38~21.47	Major gene	Kiswanto et al. 2022
Bph45	4L	Tainung71 (*O. nivara*)	—	13.70~13.80	Major gene	Li et al. 2023
qBph3	3L	IR02W101 (*O. officinalis*)	t6-f3	35.63~35.47	28%	Hu et al. 2015
qBph6	6S	IR71033-121-15	RM469-RM568	5.64~5.71	19.6%	Van Mai et al. 2015
qbph8	8L	Swarnalata	RM339-RM515	17.94~20.28	6.6%	Qiu et al. 2013
qbph11	11L	DV85	XNpb202-C1172	17.43~19.56	68.4%	Su et al. 2010
qBph4-1 (qBph4)#	4S	IR02W101 (*O. officinalis*)	P17-xc4_27	6.70~6.90	35%	Hu et al. 2015
qBph4-2 (qBph4)#	4S	BP360e	RM16382-INDEL4-5	6.20~6.70	56%	Tao et al. 2019

qBph4.2	4S	IR65482-17-511 (*O. officinalis*)	RM261-XC4-27	6.58~6.89 36–	36–44%	Hu et al. 2015
qBph4.3	4S	Salkathi	RM551-RM335	0.177~0.688	37.02%	Mohanty et al. 2017
qBph4.4	4S	Salkathi	RM335-RM5633	0.688~13.07	7.1%	Mohanty et al. 2017
qBph12	12L	ASD7	RM28466-RM7376	22.94~23.44	28.8%	Van Mai et al. 2015
Bph39(t) [bph39(t)]	—	*O. nivara*	—	—	Major gene	Akanksha et al. 2019
Bph40(t)-1 [bph40(t)]	—	*O. nivara*	—	—	Major gene	Akanksha et al. 2019

Chr: chromosome; CC-NB-LRR: coiled-coil-nucleotide-binding-leucine-rich-repeat: LRD, leucine-rich repeat domain; LRK: lectin receptor kinase; SCR: short consensus repeat.
